# Demographic and Clinical Predictors of Mortality from Highly Pathogenic Avian Influenza A (H5N1) Virus Infection: CART Analysis of International Cases

**DOI:** 10.1371/journal.pone.0091630

**Published:** 2014-03-25

**Authors:** Rita B. Patel, Maya B. Mathur, Michael Gould, Timothy M. Uyeki, Jay Bhattacharya, Yang Xiao, Nayer Khazeni

**Affiliations:** 1 Division of Pulmonary and Critical Care Medicine, Stanford University Medical Center, Stanford, California, United States of America; 2 Kaiser Permanente Southern California, Pasadena, California, United States of America; 3 Influenza Division, National Center for Immunization and Respiratory Diseases, Centers for Disease Control and Prevention, Atlanta, Georgia, United States of America; 4 Center for Health Policy and Center for Primary Care and Outcomes Research, Stanford University, Stanford, California, United States of America; 5 Department of Languages, Literatures, and Cultures, University of South Carolina, Columbia, South Carolina, United States of America; Arizona State University, United States of America

## Abstract

**Background:**

Human infections with highly pathogenic avian influenza (HPAI) A (H5N1) viruses have occurred in 15 countries, with high mortality to date. Determining risk factors for morbidity and mortality from HPAI H5N1 can inform preventive and therapeutic interventions.

**Methods:**

We included all cases of human HPAI H5N1 reported in World Health Organization Global Alert and Response updates and those identified through a systematic search of multiple databases (PubMed, Scopus, and Google Scholar), including articles in all languages. We abstracted predefined clinical and demographic predictors and mortality and used bivariate logistic regression analyses to examine the relationship of each candidate predictor with mortality. We developed and pruned a decision tree using nonparametric Classification and Regression Tree methods to create risk strata for mortality.

**Findings:**

We identified 617 human cases of HPAI H5N1 occurring between December 1997 and April 2013. The median age of subjects was 18 years (interquartile range 6–29 years) and 54% were female. HPAI H5N1 case-fatality proportion was 59%. The final decision tree for mortality included age, country, per capita government health expenditure, and delay from symptom onset to hospitalization, with an area under the receiver operator characteristic (ROC) curve of 0.81 (95% CI: 0.76–0.86).

**Interpretation:**

A model defined by four clinical and demographic predictors successfully estimated the probability of mortality from HPAI H5N1 illness. These parameters highlight the importance of early diagnosis and treatment and may enable early, targeted pharmaceutical therapy and supportive care for symptomatic patients with HPAI H5N1 virus infection.

## Introduction

Since 1997, human and poultry outbreaks of highly pathogenic avian influenza (HPAI) A (H5N1) have had devastating health, economic, and social impact in 15 countries in Asia, Africa, and the Middle East [1]–[6]. During the 2003–2004 HPAI H5N1 outbreak in Southeast Asia, for example, Vietnam culled 45 million birds at a cost of around US $118 million, and the Thai poultry industry experienced devastating economic losses of US $3 billion [4], [7].

Human cases of HPAI H5N1 virus infection with high mortality continue to be detected sporadically in several countries [8]. HPAI H5N1 patients may present with a wide range of clinical signs and symptoms, often progressing to respiratory failure and requiring invasive mechanical ventilation support [9], [10]. Human infections with HPAI H5N1 virus are associated with high mortality, but it is still largely unknown which demographic and clinical factors place an individual at higher risk of death. Studies from Hong Kong (SAR, China) [11] and Indonesia [12] have found associations between longer delays to hospitalization and increased HPAI H5N1 disease severity and mortality, but comprehensive worldwide analyses are not available. Therapeutic options include supportive care and antivirals [13]; antivirals are most effective in decreasing respiratory failure and mortality if treatment is started early [14], [15]. However, limited prognostic information is available to guide the use of scarce resources.

We aimed to statistically model individuals at highest risk of mortality from HPAI H5N1 virus infection. We systemically searched for all available data on human infections with HPAI H5N1 viruses to create a database of cases reported since the initial 1997 outbreak in Hong Kong (SAR, China). To model demographic and clinical predictors of mortality in human infection, we developed a decision tree using Classification and Regression Tree (CART) methodology [16]. These findings may help guide public health officials and policymakers in distributing limited resources.

## Methods

### Search Strategy and Inclusion Criteria

We used World Health Organization (WHO) Global Alert and Response (GAR) updates and performed systematic searches of three databases (PubMed, Scopus, and Google Scholar) to compile all confirmed and possible human cases of HPAI H5N1 virus infection. We included all articles published between January 1, 1997 and April 19, 2013 with keywords “H5N1,” “human,” and “humans.” We excluded articles that described non-human cases (animal or molecular studies), did not report individual case data, did not include data on laboratory-confirmed HPAI H5N1 cases, or described asymptomatic infections (e.g., seroprevalence studies).

We defined confirmed human H5N1 cases using the World Health Organization guidelines, requiring isolation of HPAI H5N1 virus, a positive result by reverse transcription polymerase chain reaction (RT-PCR) testing of clinical specimens using H5-specific primers and probes, an elevated H5-specific antibody titer of ≥1∶80 (or equivalent using the WHO protocol), or at least a fourfold rise in H5N1 virus neutralization antibody titer in paired sera [17]. We defined possible cases as those lacking laboratory confirmation but having symptoms and known contact with a confirmed human HPAI H5N1 case. This definition is more restrictive than the WHO definition of suspected and probable cases. The WHO definitions involve information on exposures, such as to raw poultry meat or environments contaminated with wild bird feces, that was not available to us in the literature. We then created Abstraction Form S1 based on clinical and demographic variables known to be relevant to influenza A (H1N1)pdm09 and useful in clinical practice.

We initially created a database of all cases published on the WHO Global Alert and Response (GAR) website, which includes only HPAI H5N1 cases reported from November 2003 to present. Although clinical laboratory data were not provided for each case, these were assumed to satisfy WHO reporting criteria. We then attempted to match all cases identified through literature sources to this database.

### Data Extraction

Two independent investigators (RP, MM) evaluated each article for inclusion; a third investigator (NK) resolved all disagreements. We included articles in all languages. A professional translator (YX) evaluated the numerous Chinese language articles. For Japanese, Russian, French, and Spanish languages, we verified inclusion with native-language speakers. For all other languages, we used PDF OCR X Community Edition for file conversion into text format (version 1.9.32, Burnaby, British Columbia) and a web-based translator for translation into English [18].

We then extracted the predefined set of variables for each case (Abstraction Form S1), systematically comparing demographic variables to avoid duplication. One co-author (TU) reviewed discrepancies between the WHO GAR publications and the published medical literature; based on his familiarity with several of these cases, we resolved discrepancies using data from the literature.

### Variables

Using our pre-defined Abstraction Form S1, we extracted our primary outcome variable (mortality), demographic predictor variables (age, sex, country, per capita government expenditure on health [PCGEH], season, body mass index [BMI], and comorbidities), infection-related predictor variables (contact with poultry, delay from symptom onset to hospitalization, and whether the case was part of a cluster of known cases), and hospitalization predictor variables (laboratory data, pneumonia, acute respiratory distress syndrome [ARDS], and mechanical ventilation). We defined a case cluster as at least two geographically and temporally proximal (epidemiologically-linked) confirmed human HPAI H5N1 cases.

We obtained PCGEH at the average exchange rate (USD) for each country and year through the World Health Organization Global Health Observatory Data Repository [19]. Since data beyond 2011 are unavailable, data from 2011 were carried forward for later cases. Finally, we created a season variable based on month (Summer: June–August; Fall: September–November; Winter: December–February; Spring: March–May).

### Statistical Analysis

We performed all statistical analyses using R software (Version 3.0.0, Vienna, Austria) and defined statistical significance by an alpha level of 0.05. Our primary analytic goal was to develop a parsimonious decision tree model with optimal predictive ability for mortality following HPAI H5N1 virus infection. We first assessed bivariate associations between each predictor variable and mortality using logistic regression models. For continuous predictors (age, delay from hospitalization to symptom onset, and PCGEH), we visually assessed for the linearity assumption (Figure S1).

All continuous predictor variables had potentially nonlinear relationships with death, so we analyzed them both in continuous and in categorical form. We divided age into four categories similar to those associated with mortality from influenza A (H1N1)pdm09: 0–4 years, >4–18 years, >18–25 years, and >25 years [20]. PCGEH was split in quartiles. We created three groups of countries because most countries had very few cases; including these countries as individual predictors would cause over-fitting and statistical instability [21]. We therefore defined three categories of countries: Indonesia (n = 171), Egypt (n = 169), and all others combined (“Other”). Fewer than 100 cases were documented in each of the countries in the “Other” category.

As we performed initial analyses, we found that several parameters were missing data. We therefore developed a decision tree using CART methods [16]. CART models generally yield comparable results to logistic regression models, but unlike logistic regression, CART methods do not require missing data to be deleted or imputed (minimizing possible bias), capture higher-order interactions more easily, do not assume an underlying linear model, and generate a graphical prediction tool that is easy for practitioners to use and interpret [22], [23].

CART procedures build a decision tree by selecting locally optimal splits that minimize “impurity” on the outcome measure of the two child nodes. Low impurity on the outcome measure indicates that the classifier performs well at separating observations with one outcome (e.g., death) from observations with another outcome (e.g., survival). For example, if sex is a strong risk factor for mortality, then mortality will be similar within each sex and different between sexes. All possible binary splits are considered for both continuous and categorical variables. The initial split is chosen as the single best classifier on the outcome measure; then, within each child node, the splitting procedure is recursively repeated until no further splits are possible. All observations, including those with missing data, are included in model-building: at each split, the impurity index is simply calculated over only those observations not missing the relevant predictor variable. To avoid over-fitting, the initial large tree is pruned based on a cost-complexity index, which captures the tradeoff between better fit and added complexity due to each additional node in the tree [21]. The optimal final tree, defined as the tree with the lowest expected misclassification, is selected using cross-validation [16].

We assessed model performance using a receiver operating characteristic (ROC) curve and corresponding area under the curve (AUC) [23]. We constructed bootstrapped confidence intervals for the sensitivity and specificity thresholds of the ROC curve and for the AUC. To replicate the results of the CART model, we developed corresponding prognostic models using complete-case logistic regression (Table S1) and multiply-imputed logistic regression (Table S2).

## Results

### Report Identification and Eligibility

**Figure 1:**
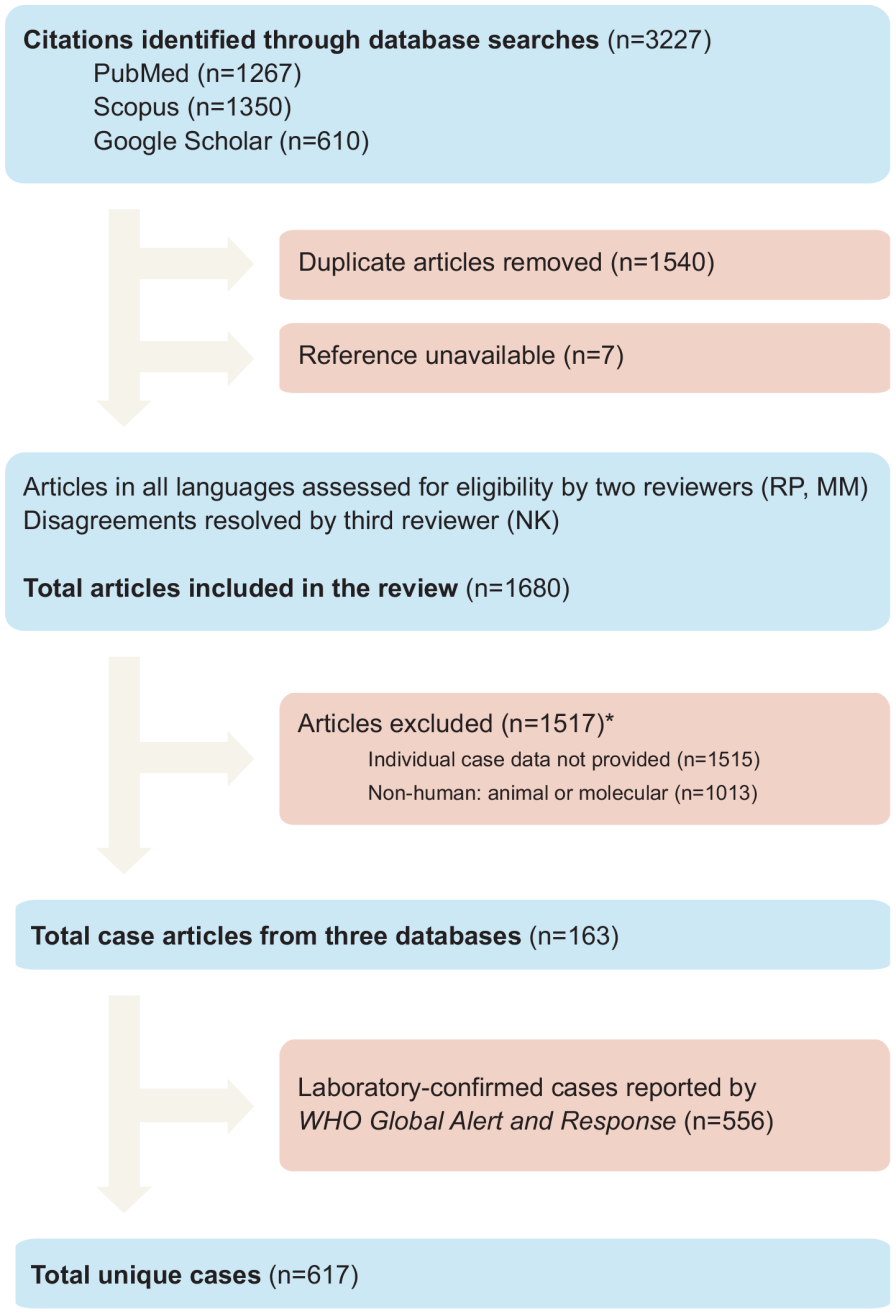
Literature search strategy. *: Total number of excluded articles is less than the sum of articles excluded by each criterion because most articles failed multiple criteria.

Our search identified 3,227 potentially relevant articles published since 1997 (Figure 1). After removing 1,540 duplicate articles and 7 unavailable articles, two investigators (RP, MM) independently reviewed the remaining 1,680 articles. The review process yielded 163 articles meeting inclusion criteria, comprising 617 unique cases. Nearly all studies meeting inclusion criteria were case reports, so we did not assess methodological quality criteria.

### Data Quality

**Figure 2:**
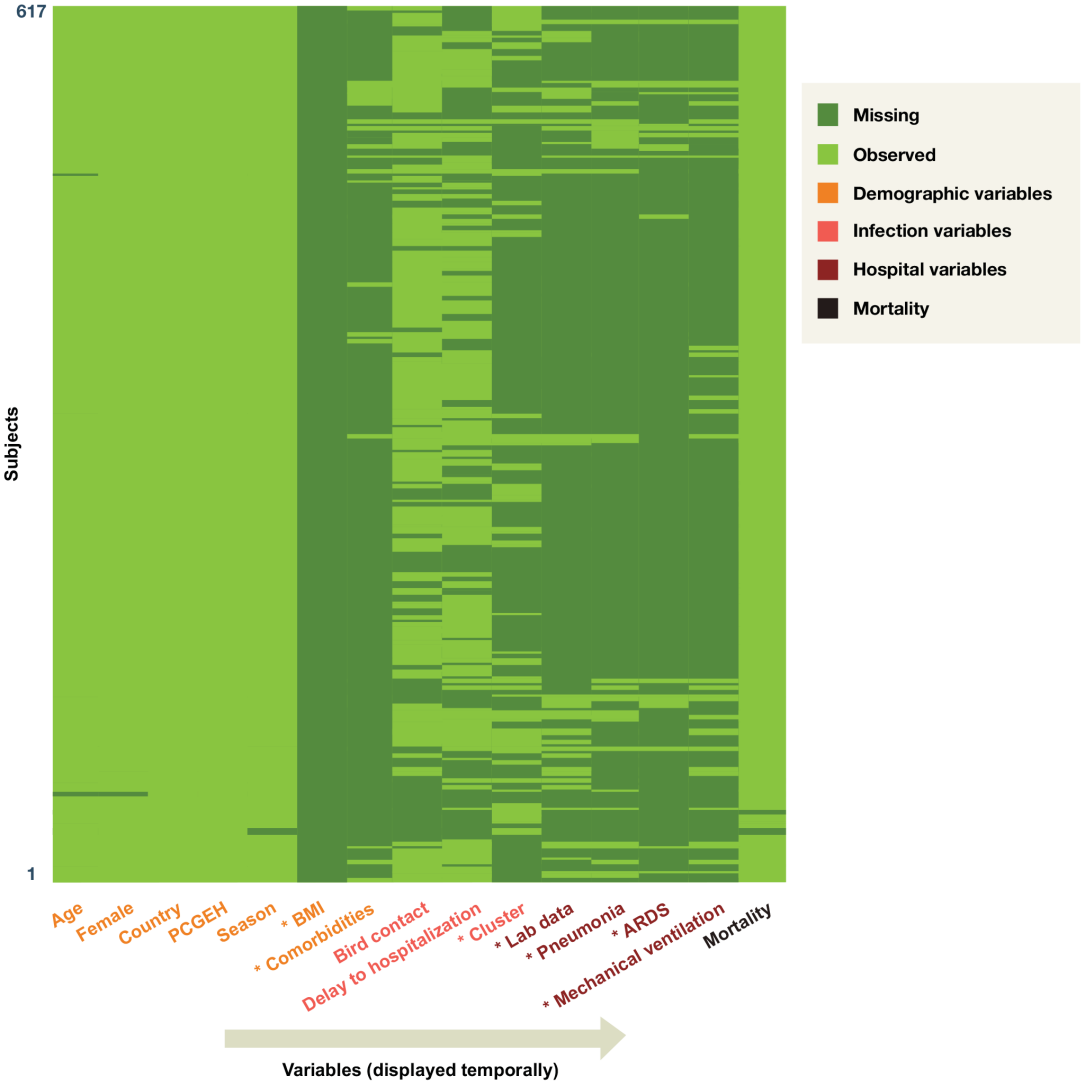
Variable summary and patterns of missing data. *: Variable was excluded from modeling. Each row represents one of 617 human cases; each column represents a variable abstracted from the literature. The color of each cell indicates whether the corresponding variable was missing (dark green) or observed (light green) for the given case.

The quality of data reporting on HPAI H5N1 cases was inconsistent. Several variables had missing data, most with homogeneity among the non-missing values; these were not included in the analysis (Figure 2). In other words, BMI, presence of comorbidities, whether the case was part of a cluster, laboratory data, presence of pneumonia, ARDS, and use of mechanical ventilation were mentioned almost exclusively for cases in which the parameter was present rather than absent. For example, only 10 case reports noted that a case *had not* received mechanical ventilation, 63 noted that a case *had* received ventilation, and the remaining 544 offered no information. In light of this limitation of the case-reporting process, we narrowed the scope of our prognostic model to include only demographic and infection variables, as post-admission hospital variables were usually unavailable. We removed these variables from analysis because we would have limited statistical power to assess their effects, and infrequently recorded homogenous variables may have little clinical utility (see Figure 2 for details on excluded variables). The eight variables used in analysis had 9% missing observations.

### Participant Characteristics

Demographic and clinical characteristics of the 617 cases are presented in Table 1. Overall mortality was 59%. Cases occurred in 15 countries, with 55% reported from Indonesia and Egypt combined (n = 171 and n = 169, respectively). 140 cases occurred within epidemiologically- and geographically-linked clusters, 13 occurred sporadically, and cluster status was unknown for the remaining 464. Cases tended to be young adults (median age 18 years; IQR 6–29). There was a marginal predominance of females (54% female, chi-square = 3.75, df = 1, p = 0.05). For all demographic parameters reported in the WHO GAR case summary (year, country, and mortality) [8], distributions for our literature-based dataset and the WHO summary were nearly identical (Table 1). We find little indication, therefore, of reporting or selection bias in our sample.

**Table 1:** Demographic and clinical characteristics of study sample.

Characteristic	Literature search (n = 617)	Reported WHO HPAI H5N1 cases as of Oct 31, 2013 [8], [48]–[50] (n = 644)
**Year**		
1997	24 (4%)	*not reported*
1998	4 (0.6%)	*not reported*
2003	7 (1%)	4 (0.6%)
2004	53 (9%)	46 (7%)
2005	75 (12%)	98 (15%)
2006	116 (19%)	115 (18%)
2007	90 (15%)	88 (14%)
2008	45 (7%)	44 (7%)
2009	53 (9%)	73 (11%)
2010	48 (8%)	48 (7%)
2011	62 (10%)	62 (10%)
2012	29 (5%)	32 (5%)
2013	10 (2%)	34 (5%)
Missing	1 (0.1%)	
**Country**		
Indonesia	171 (28%)	194 (30%)
Egypt	169 (27%)	173 (27%)
Vietnam	96 (16%)	125 (19%)
China	50 (8%)	45 (7%)
Cambodia	36 (6%)	44 (7%)
Hong Kong (SAR, China)	29 (5%)	*not reported*
Thailand	27 (4%)	25 (4%)
Turkey	11 (2%)	12 (2%)
Azerbaijan	9 (1%)	8 (1%)
Bangladesh	6 (1%)	7 (1%)
Pakistan	5 (0.8%)	3 (0.5%)
Iraq	3 (0.5%)	3 (0.5%)
Laos	2 (0 3%)	2 (0.3%)
Djibouti	1 (0.2%)	1 (0.2%)
Myanmar	1 (0.2%)	1 (0.2%)
Nigeria	1 (0.2%)	1 (0.2%)
**PCGEH (USD)**	24.8 (13.7–49.0)	*not reported*
**Sex**		
Female	331 (54%)	*not reported*
Male	283 (46%)	
Missing	3 (0.5%)	
**Age** (yrs)	18 (6–29)	*not reported*
Missing	9 (1%)	
**Season**		
Summer	64 (10%)	*not reported*
Fall	85 (14%)	
Winter	285 (46%)	
Spring	177 (29%)	
Missing	6 (1%)	
**Delay to hospitalization from symptom onset (days)**	4 (2–6)	*not reported*
Missing	242 (39%)	
**Contact with poultry**		
Yes	356 (58%)	*not reported*
Likely yes	62 (10%)	
No	20 (3%)	
Missing	179 (29%)	
**Mortality**		
Death	362 (59%)	382 (59%)
Survival	245 (40%)	262 (41%)
Missing	10 (2%)	

*Data are frequency (%) or median (first quartile – third quartile).*

*Percentages are calculated including missing observations.*

*PCGEH = per capita government expenditure on health.*

### Bivariate Associations with Mortality

In bivariate logistic regression models, risk factors for mortality were longer delay to hospitalization, infection not in Egypt, older age, lower PCGEH, likely contact with poultry, female sex, and illness onset during summer months (Table 2).

**Table 2:** Bivariate associations of candidate predictor variables with mortality.

Variable		Survived (n = 245)	Died (n = 362)	Odds ratio (95% CI)	p value coefficient	p value model	c-statistic
**Delay to hospitalization**		3 (1–5)	5 (3–6)	1.31 (1.20, 1.45)	<0.0001	<0.0001	0.70
**Country**	Egypt	108 (64%)	61 (36%)	*Ref*	*Ref*		
	Indonesia	30 (18%)	141 (82%)	8.32 [5.08, 13.95]	<0.0001	<0.0001	0.69
	Other	107 (40%)	160 (60%)	2.65 [1.78, 3.96]	<0.0001		
**Age group**	0–4	89 (74%)	32 (26%)	0.14 [0.07, 0.25]	<0.0001		
	>4–18	64 (33%)	128 (67%)	0.75 [0.42, 1.32]	0.33	<0.0001	0.65
	>18–25	23 (27%)	61 (73%)	*Ref*	*Ref*		
	>25	66 (33%)	136 (67%)	0.78 [0.44, 1.35]	0.38		
**Age**		9.5 (3–27)	20 (12–30)	1.03 [1.02, 1.04]	<0.0001	<0.0001	0.64
**PCGEH group**	<13.7	62 (41%)	89 (59%)	*Ref*	*Ref*		
	>13.7–24.8	31 (20%)	122 (80%)	2.74 [1.66, 4.61]	0.0001	<0.0001	0.63
	>24.8–49.0	84 (52%)	77 (48%)	0.64 [0.41, 0.998]	0.05		
	>49.0	68 (48%)	74 (52%)	0.76 [0.48, 1.20]	0.24		
**Contact with poultry**	No	10 (53%)	9 (47%)	*Ref*	*Ref*		
	Yes	174 (49%)	182 (51%)	1.16 [0.46, 2.99]	0.75	<0.0001	0.59
	Likely yes	8 (13%)	54 (87%)	7.50 [2.38, 25.15]	0.0007		
**PCGEH**		35.1 (13.7–49.1)	23.2 (16.4–38.0)	0.994 [0.989, 0.998]	0.007	0.004	0.59
**Sex**	Male	134 (55%)	147 (45%)	*Ref*	0.0007	0.0007	0.57
	Female	110 (41%)	213 (59%)	1.77 [1.27, 2.45]			
**Season**	Summer	17 (27%)	47 (73%)	*Ref*	*Ref*		
	Fall	37 (44%)	48 (56%)	0.47 [0.23, 0.94]	0.03	0.02	0.57
	Winter	106 (38%)	174 (62%)	0.59 [0.32, 1.07]	0.09		
	Spring	84 (47%)	93 (53%)	0.40 [0.21, 0.74]	0.004		

*Data are presented as medians (IQR) or frequencies (%). Variables are ordered roughly by statistical significance. Row percentages are calculated excluding missing data. P-values were calculated using Wald's z-test for logistic regression coefficients and the likelihood-ratio test for regression models.*

*PCGEH = per capita government expenditure on health.*

### Decision Tree Model for Mortality

**Figure 3:**
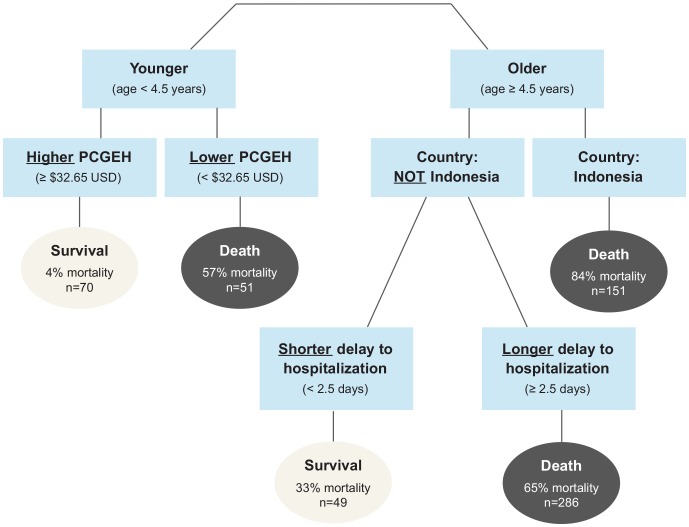
Classification tree for mortality following highly pathogenic avian influenza H5N1 virus infection. Model was trained on all n = 607 cases with observed mortality. The following variables were candidates for inclusion: age, PCGEH, country, delay to hospitalization, sex, season, contact with poultry.

The decision tree, trained on all 607 cases with observed mortality, evaluated seven candidate predictor variables: age, PCGEH, country group, delay from symptom onset to hospitalization, sex, contact with poultry, and season. The variables are listed here in descending order of variable importance in the decision tree, a measure based on split quality. The first four were used as splitting variables in the final, pruned tree (Figure 3).

The first node splits on age, with higher mortality in patients at least 4.5 years of age. In the second level of the tree, young children (<4.5 years) in high-PCGEH settings (≥32.65 USD) are predicted to survive, with the lowest mortality (4%) of all groups. Young children in low-PCGEH settings (<32.65 USD) are predicted to die (57% mortality).

Older patients (≥4.5 years) are further partitioned by country. Older cases in Indonesia are predicted to die, with the highest mortality (84%) of all groups. Older cases not in Indonesia are classified by one final split based on delay to hospitalization: cases with a short delay to hospitalization (<2.5 days) are predicted to survive (33% mortality), while cases hospitalized later after illness onset (≥2.5 days) are predicted to die (65% mortality).

### Model Performance

**Figure 4:**
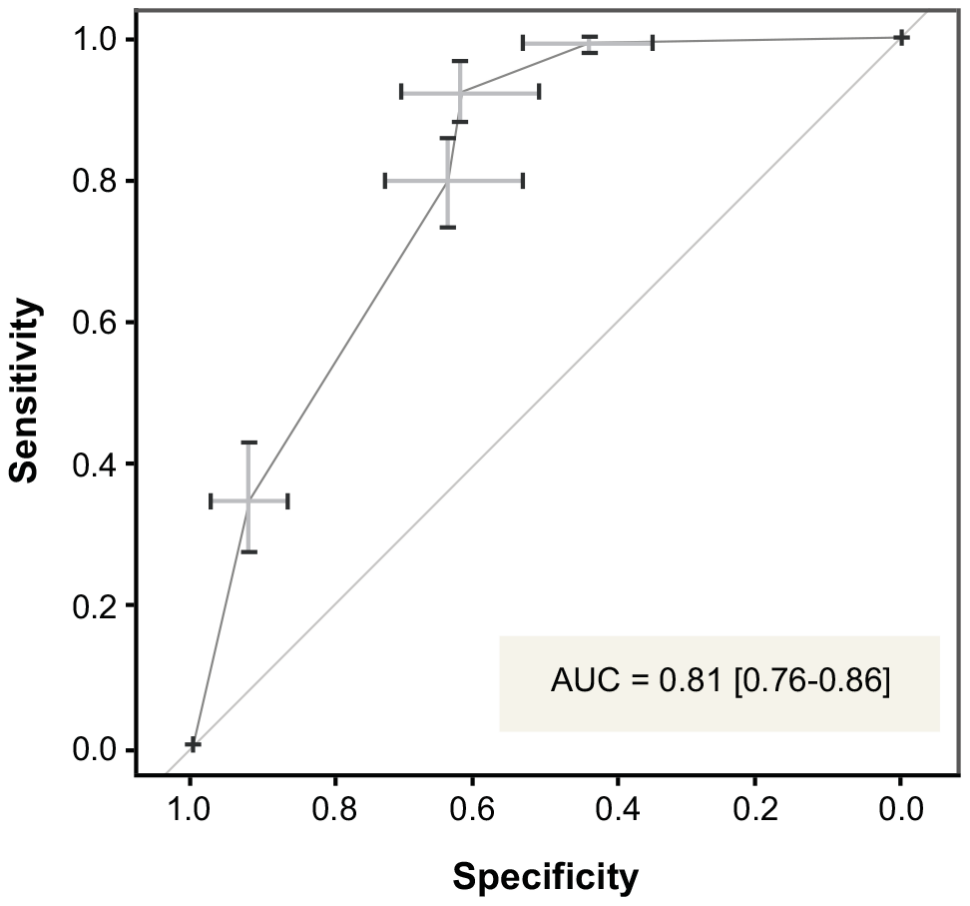
ROC curve for pruned CART tree. ROC curve represents performance of CART model on all cases without missing observations on any model variables (n = 301). Error bars represent bootstrapped 95% confidence intervals for sensitivity-specificity thresholds.

We assessed the decision tree's performance with an ROC curve and corresponding AUC (Figure 4). Tested on all cases without missing data on mortality or any of the seven candidate model variables (n = 301), the AUC was 0.81 (95% CI: 0.76–0.86), indicating very good discrimination. Performance remained strong (AUC = 0.75; 95% CI: 0.71–0.78) when the model was instead tested on all cases with observed outcome but potentially missing any subset of the predictors (n = 607). The latter is a more difficult prediction task, requiring surrogate splitting based on the missing predictors. In surrogate splitting, the value of the missing predictor is estimated using the other predictors. This estimated value is then used as usual to classify the observation.

### Sensitivity Analysis

We also performed complete-case logistic regression and multiply-imputed logistic regression including all predictor variables that were candidates for inclusion in the CART model. All models yielded similar results (Tables S1 and S2).

## Discussion

We investigated factors associated with increased mortality following HPAI H5N1 virus infection to guide public health messages, resource distribution, and triage of infected individuals. We conducted a systematic search of all available literature describing human cases of HPAI H5N1 virus infection and developed a prognostic decision tree. We find that age, health expenditure, delay from symptom onset to hospitalization, and country are significant predictors of mortality. Additionally, we find that data reporting is incomplete and poorly standardized.

Our finding that HPAI H5N1 mortality is lowest in young children aged 0 to 4.5 years is different from established patterns observed for seasonal influenza in which mortality is high in infants and young children [24], [25]. Cultural influences may contribute to lower mortality from HPAI H5N1 in young children: in Egypt, for example, parents tend to seek medical care for ill children promptly, and pediatric cases tend to receive earlier hospitalization and treatment with oseltamivir [26]. For seasonal influenza, elderly persons are at the highest risk of mortality of all age groups [27]. However, we could not determine if HPAI H5N1 in humans parallels this pattern because the literature contained only four case reports in individuals aged 65 years or older. The rarity of HPAI H5N1 cases among elderly may reflect less frequent exposure to poultry or other, unknown factors.

Not surprisingly, we found that reduced national healthcare expenditure is associated with higher mortality from HPAI H5N1. This relationship is common with many diseases at a country-specific level. We were unable to delineate the complete mechanisms responsible for this finding, but healthcare quality may be a mediator. Maternal mortality, generally considered a sensitive indicator of overall quality and accessibility of healthcare, is widely discrepant across affected countries in our study. For example, maternal mortality per 100,000 live births is 55 in China, 95 in Vietnam, and 440 in Cambodia [28].

We also find that a longer delay from HPAI H5N1 illness onset to hospitalization is associated with higher mortality, a finding previously reported in smaller, geographically restricted datasets [11], [12], [26], [29]. The reason for this is unclear but may be related to delayed administration of antiviral treatment, associated in observational analyses with higher mortality [12], [14], [15]. Ferrets experimentally inoculated with HPAI H5N1 viruses and treated early with oseltamivir had significantly reduced clinical symptoms and mortality [30], [31]. We did not have access to detailed data on antiviral treatment; however, our results support public health messages in countries with endemic HPAI H5N1 or periodic poultry outbreaks recommending prompt antiviral treatment and H5N1 testing for symptomatic persons with a recent history of poultry contact.

Consistent with WHO cumulative case counts and previously published analyses [32], we found that Indonesian cases have very high mortality (82%). This may be secondary to a combination of unknown factors, as well as several of the other risk factors we identified occurring more frequently in Indonesian cases. Such confounding cannot be adequately resolved with either CART or standard multiple regression procedures [33]. As noted above, Indonesia has limited healthcare resources (demonstrated by 380 maternal deaths per 100,000 live births in Indonesia compared to 55 and 170, respectively, in China and Egypt [28]). Additionally, physician density per 1,000 population is much lower in Indonesia (0.13) than in Egypt (0.54) and China (1.64), the other two countries with the highest number of HPAI H5N1 cases [28]. HPAI H5N1 cases may be recognized late in the clinical course and therefore treated with antivirals relatively late; previous analyses of Indonesian cases found a median time from symptom onset to treatment with oseltamivir of 7 days [12], [32]. Our findings again support prioritizing public health messages urging early medical attention in case of influenza signs and symptoms, especially for individuals in Indonesia.

We were surprised to find inconsistent case reporting in the literature. While the WHO maintains summary data on worldwide cases of HPAI H5N1, this aggregation provides minimal individual-level demographic and clinical characteristics [8]. The detailed WHO Clinical Case Summary Form for reporting human HPAI H5N1 cases [34] is rarely used in practice [35]. Additionally, reporting and surveillance practices can differ greatly by country and locality [35], [36]. Relevant clinical factors such as the presence of co-morbidities and use of mechanical ventilation are mentioned almost exclusively for cases in which the parameter was present rather than absent, and important demographic factors, such as place of residence (urban vs. rural), are rarely noted [37].

Reporting bias may exist; practitioners may consider a diagnosis of HPAI H5N1 only for more severe cases, limiting reports of subclinical or asymptomatic infections in the medical literature. Controversy exists over the extent to which such a bias may inflate HPAI H5N1 mortality estimates. A meta-analysis reported an average seroprevalence of HPAI H5N1 virus antibodies of 1 to 2%, potentially translating into a substantial number of unreported cases worldwide [38]; however, others have criticized these findings for the use of non-representative, high-risk populations [39]. One review included 29 serologic studies and found no clear serological evidence of “mild” HPAI H5N1 virus infections [40]. However, some studies have reported serologic evidence of rare, sporadic asymptomatic or clinically mild HPAI H5N1 virus infection [41]–[43]. Although more research is required, large numbers of mild, unreported HPAI H5N1 cases appear unlikely.

Potential methodological limitations include variability in surveillance and clinical care, lack of data on antiviral treatment, and time from illness onset to start of antiviral treatment. Additionally, time from onset to hospitalization may not equal time to oseltamivir treatment onset. We noted a high proportion of unreported variables. Complete-case analysis can cause bias and imprecision in regression coefficient estimates, particularly if data are not missing at random. Nevertheless, we performed three analytic techniques with fundamentally different approaches to handling missing data, and they all yielded similar results. Like all classification trees, our model demonstrates some statistical instability [21]. This arises due to the hierarchical nature of node splitting, whereby small changes in the training data can change the splits and resulting tree. Since statistical methods to improve stability, such as bagging, generally have the disadvantage of obscuring the classification procedure from interpretation [21], we opted to optimize interpretability and stability with a conservatively pruned decision tree. Additionally, we were unable to assess the role of host genetic factors, which have been postulated to increase the risk of severe influenza disease [44]–[47].

From a policy standpoint, improved recognition of disease (albeit rare) and early delivery of healthcare, especially antiviral treatment, could result in reduced hospitalization costs, decreased morbidity, and lower mortality from HPAI H5N1 virus infection. To facilitate analyses, the idiosyncratic case reporting process our study detected could be greatly improved by widespread adoption of a standardized data collection form, such as an online database. Currently, the WHO receives case report data from officials at Ministries of Health, which collect case report data from local hospitals. A convenient and efficient mode of data collection may enable improved communication at both reporting junctures.

We have established a predictive classification tree model to estimate human HPAI H5N1 mortality based on readily available clinical and demographic predictors: age, delay from symptom onset to hospitalization, country, and PCGEH. Our resulting publicly accessible online algorithm (http://flubusters.stanford.edu) may allow public health officials and clinicians to triage patients and distribute limited resources. To our knowledge, our work is the most complete literature search and analysis of worldwide human cases of HPAI H5N1. Contingent on improved data collection, future research should investigate the predictive ability of clinical and demographic characteristics not currently available in the case literature. Improved reporting and predictive strategies are essential, particularly in light of recent research [45]–[47] that suggests only a few virus mutations may increase the risk of human-to-human HPAI H5N1 virus transmission.

## Supporting Information

Abstraction Form S1 (PDF)

Figure S1 **Probability of death conditional on continuous predictors.** Error bars (± SE) are based on the binomial distribution.
(EPS)

Table S1 **Complete-cases logistic regression.**
(PDF)

Table S2 **Multiply-imputed logistic regression (m = 5 imputations).**
(PDF)
