# Supplementary material for: Demographic and Clinical Predictors of Mortality from Highly Pathogenic Avian Influenza A (H5N1) Virus Infection: CART Analysis of International Cases
Source: PLoS One. 2014 Mar 25;9(3):e91630. doi: 10.1371/journal.pone.0091630 (PMC3965392; doi:10.1371/journal.pone.0091630)
Supplement: Abstraction Form S1 — (PDF) [file pone.0091630.s001.pdf]

Abstraction form S1

ABSTRACTION INFORMATION

Reviewer **Date:** ________________

- ☐ MM
- ☐ RP
- ☐ NK

CITATION INFORMATION

Search Engine: ____________________ **Reference #:** ____________________________

Title: ____

Journal: ____

Year: ________

Authors: ____

Institution(s): ____

Relevant References: ____

____

____

LANGUAGE

- ☐ English
- ☐ Other (describe language and translator): ____

ARTICLE INCLUSION/EXCLUSION RATIONALE

Does this article meet inclusion criteria?

YES. Requires all categories to meet criteria:

- ☐ Human subject
- ☐ Individual case data available (including meta-analysis articles)
- ☐ Laboratory-confirmed influenza A (H5N1) virus OR suspected H5N1 case amongst known case cluster.
  - H5N1 culture *or*
  - H5N1 PCR study *or*
  - Antibody titer 1:80

NO. Does not meet criteria for these reasons:

- ☐ Non-human: molecular or animal
- ☐ Individual case details not provided
- ☐ Insufficient epidemiological data

COMPLETE THE FOLLOWING IF THE ARTICLE MET INCLUSION CRITERIA:

STUDY DESIGN INFORMATION

Describe the study design:

PRE-ADMISSION PREDICTOR VARIABLES DESCRIBED

Demographic variables:

- ☐ Country where A (H5N1) virus infection identified
- ☐ Per capita government expenditure on health (PCGEH)
- ☐ Season
- ☐ Age
- ☐ Sex
- ☐ Body Mass Index (BMI)
- ☐ Co-morbidities

Infection-related variables:

- ☐ Case is part of a cluster of known cases
- ☐ Contact with poultry prior to symptom onset
- ☐ Delay from symptom onset to hospitalization (days)

POST-ADMISSION PREDICTOR VARIABLES DESCRIBED

Hospitalization variables:

- ☐ Laboratory data
- ☐ Presence of pneumonia
- ☐ Acute respiratory distress syndrome (ARDS)
- ☐ Mechanical ventilation

OUTCOME VARIABLES DESCRIBED

- ☐ Mortality

ADDITIONAL COMMENTS
