# Supplementary material for: Demographic and Clinical Predictors of Mortality from Highly Pathogenic Avian Influenza A (H5N1) Virus Infection: CART Analysis of International Cases
Source: PLoS One. 2014 Mar 25;9(3):e91630. doi: 10.1371/journal.pone.0091630 (PMC3965392; doi:10.1371/journal.pone.0091630)
Supplement: Table S1 — Complete-cases logistic regression. (PDF) [file pone.0091630.s003.pdf]

***Table S1:* Complete-cases logistic regression**

**Variable**		**Odds ratio (95% CI)**	**p value coefficient**
Age		1.05 [1.03, 1.07]	<0.0001
Country	Egypt	*Ref*	*Ref*
	Indonesia	11.54 [4.65, 31.01]	<0.0001
	Other	3.78 [1.90, 7.67]	0.0002
Delay to hospitalization		1.17 (1.04, 1.32)	0.01
PCGEH		0.99 [0.99, 1.00]	0.06
Sex	Male	*Ref*	*Ref*
	Female	1.68 [0.93, 3.05]	0.09
Contact with poultry	No	*Ref*	*Ref*
	Yes	2.20 [0.51, 8.89]	0.27
	Likely yes	4.93 [0.87, 28.06]	0.07
Season	Summer	*Ref*	*Ref*
	Fall	0.32 [0.10, 0.96]	0.05
	Winter	1.00 [0.36, 2.67]	0.99
	Spring	0.84 [0.30, 2.34]	0.74

Model trained on cases without missing data on included variables or mortality (n=301).
Coefficient p-values were calculated using Wald's z-test.
This model includes all predictors included in the CART model.
