# Supplementary material for: Demographic and Clinical Predictors of Mortality from Highly Pathogenic Avian Influenza A (H5N1) Virus Infection: CART Analysis of International Cases
Source: PLoS One. 2014 Mar 25;9(3):e91630. doi: 10.1371/journal.pone.0091630 (PMC3965392; doi:10.1371/journal.pone.0091630)
Supplement: Table S2 — Multiply-imputed logistic regression (m = 5 imputations). (PDF) [file pone.0091630.s004.pdf]

***Table S2:* Multiply-imputed logistic regression (m=5 imputations)**

Variable		Odds ratio (95% CI)	p value coefficient
Age		1.03 [1.01, 1.04]	0.0005
Country	Egypt	*Ref*	*Ref*
	Indonesia	5.31 [2.79, 10.12]	<0.0001
	Other	2.28 [1.41, 3.68]	0.0008
Delay to hospitalization		1.23 [1.06, 1.43]	0.01
PCGEH		1.00 [0.99, 1.00]	0.37
Sex	Male	*Ref*	
	Female	1.80 [1.22, 2.66]	0.003
Contact with poultry	No	*Ref*	*Ref*
	Yes	1.77 [0.65, 4.81]	0.25
	Likely yes	4.03 [1.12, 14.49]	0.03
Season	Summer	*Ref*	*Ref*
	Fall	0.34 [0.15, 0.78]	0.01
	Winter	0.61 [0.29, 1.28]	0.19
	Spring	0.55 [0.25, 1.18]	0.12

Model trained on all n=617 cases with m=5 imputations.
Coefficient p-values were calculated using Wald’s z-test.
This model includes all predictors included as candidates in the CART model.
